# Visual Population Receptive Fields in People with Schizophrenia Have Reduced Inhibitory Surrounds

**DOI:** 10.1523/JNEUROSCI.3620-15.2016

**Published:** 2017-02-08

**Authors:** Elaine J. Anderson, Marc S. Tibber, D. Sam Schwarzkopf, Sukhwinder S. Shergill, Emilio Fernandez-Egea, Geraint Rees, Steven C. Dakin

**Affiliations:** ^1^Institute of Ophthalmology,; ^2^Institute of Cognitive Neuroscience,; ^3^Wellcome Trust Centre for Neuroimaging, and; ^4^Experimental Psychology, University College London, London, United Kingdom,; ^5^Department of Psychosis Studies, Institute of Psychiatry, Psychology and Neuroscience, King's College London, London, United Kingdom,; ^6^South London and Maudsley National Health Service Foundation Trust, London, United Kingdom,; ^7^Clozapine Clinic, Cambridgeshire and Peterborough National Health Service Foundation Trust, and; ^8^Department of Psychiatry, Behavioural and Clinical Neuroscience Institute, University of Cambridge, Cambridge, United Kingdom, and; ^9^School of Optometry and Vision Science, University of Auckland, Auckland, New Zealand

**Keywords:** fMRI, perception, pRF, schizophrenia, surround suppression, visual cortex

## Abstract

People with schizophrenia (SZ) experience abnormal visual perception on a range of visual tasks, which have been linked to abnormal synaptic transmission and an imbalance between cortical excitation and inhibition. However, differences in the underlying architecture of visual cortex neurons, which might explain these visual anomalies, have yet to be reported *in vivo*. Here, we probed the neural basis of these deficits using fMRI and population receptive field (pRF) mapping to infer properties of visually responsive neurons in people with SZ. We employed a difference-of-Gaussian model to capture the center-surround configuration of the pRF, providing critical information about the spatial scale of the pRFs inhibitory surround. Our analysis reveals that SZ is associated with reduced pRF size in early retinotopic visual cortex, as well as a reduction in size and depth of the inhibitory surround in V1, V2, and V4. We consider how reduced inhibition might explain the diverse range of visual deficits reported in SZ.

**SIGNIFICANCE STATEMENT** People with schizophrenia (SZ) experience abnormal perception on a range of visual tasks, which has been linked to abnormal synaptic transmission and an imbalance between cortical excitation/inhibition. However, associated differences in the functional architecture of visual cortex neurons have yet to be reported *in vivo*. We used fMRI and population receptive field (pRF) mapping to demonstrate that the fine-grained functional architecture of visual cortex in people with SZ differs from unaffected controls. SZ is associated with reduced pRF size in early retinotopic visual cortex largely due to reduced inhibitory surrounds. An imbalance between cortical excitation and inhibition could drive such a change in the center-surround pRF configuration and ultimately explain the range of visual deficits experienced in SZ.

## Introduction

People with schizophrenia (SZ) experience abnormal visual perception on a range of visual tasks (Butler et al., 2008), including reduced contrast sensitivity (Slaghuis, 1998) poor orientation discrimination (Tibber et al., 2015), impaired motion processing (Chen et al., 2004; Kim et al., 2006), and anomalous global processing such as detecting contours embedded in noise (Silverstein et al., 2000; Uhlhaas et al., 2006b; Robol et al., 2013). These findings have been interpreted as a general failure of integration, such that objects and scenes are experienced as fragmented parts rather than coherent wholes (Silverstein and Keane, 2011).

People with SZ are also less influenced by visual context such that their visual discrimination performance is less affected by the presence of disruptive (Tadin et al., 2006) or facilitatory (Must et al., 2004) surrounds and they experience weaker illusions based on visual context compared with unaffected observers (Dakin et al., 2005; Robol et al., 2013; Tibber et al., 2013; Yang et al., 2013). For example, in the “contrast–contrast” illusion, a typical observer would perceive a target patch to be of higher contrast when placed in isolation compared with when embedded within a high-contrast surround (Chubb et al., 1989). People with SZ experience weaker contrast–contrast effects that result in more accurate judgements of target contrast (Dakin et al., 2005; Tibber et al., 2013; Yang et al., 2013; Schallmo et al., 2015), although effect size seems to depend on symptom severity (Barch et al., 2012). These findings suggest that a specific neural mechanism differs in this group, rather than poor comprehension or performance of the task. Such “surround suppression” is thought to be mediated by the inhibition of a neuron's response to a stimulus by the pooled activity of cells in the surrounding cortex (Cavanaugh et al., 2002). For simple grating stimuli, this has been shown to operate within V1, V2, and V3, with the strongest effects in V2 and V3 (Zenger-Landolt and Heeger, 2003).

Tests of visual perception are becoming popular for developing models of impaired neural processing in SZ (Silverstein and Keane, 2011; Metzner et al., 2014; Phillips et al., 2015; Silverstein et al., 2015). For example, the neurodevelopmental hypothesis posits that SZ results from disturbed neural development (Murray and Lewis, 1987; Weinberger, 1987) that could affect the neural architecture of visual cortex (with knock-on effects for perception). Perceptual deficits in SZ have often been linked to a selective loss of large magnocellular (M-cell) neurons (Butler et al., 2005; Martínez et al., 2008), with correspondingly large receptive fields that respond preferentially to coarse-scale dynamic image structure. Their loss could account for poor sensitivity to low SFs (Butler et al., 2001) and impaired motion processing in SZ (O'Donnell et al., 1996; Chen et al., 2004; Kim et al., 2006). Consistent with this, postmortem studies have indicated a reduction in mean neuronal somal size (Rajkowska et al., 1998), but also increased neuronal density (Selemon et al., 1995) and a reduction in neuron number, volume, and surface area of V1 in SZ (Dorph-Petersen et al., 2007).

To date, *in vivo* neuroimaging has provided mixed reports on whether the magnitude of responses and/or the topography of early visual areas differ between patients with SZ and controls (Martínez et al., 2008; Wynn et al., 2008). Wynn et al. (2008) found good spatial overlap of normalized retinotopic maps in early visual areas in people with SZ and healthy controls and no difference in peak response amplitude, whereas Martínez et al. (2008) found the cortical extent of V1 and V2 to be 15% lower in SZ. However, both of these studies determined functional properties of the visual cortex at a macroscopic spatial scale (e.g., retinotopic maps). Here, we sought to clarify whether the fine-grained functional architecture of visual cortex in people with SZ differs from unaffected controls using fMRI and population receptive field (pRF) mapping (Wandell et al., 2007; Dumoulin and Wandell, 2008; Wandell and Winawer, 2015) to estimate the size (width) of pRFs in retinotopic visual cortex. In addition, by using a difference of Gaussian (DoG) model, we probed the spatial scale of the pRFs inhibitory surround (Zuiderbaan et al., 2012). First, we hypothesized that, if SZ is associated with a selective loss of M-cells, then we would observe a bias toward smaller pRF size in patients compared with controls. Second, we hypothesized that the center-surround relationship would differ in people with SZ compared with controls such that the inhibitory surround would be reduced (in size and/or depth), providing a possible explanation for the reduced surround suppression observed in this group.

## Materials and Methods

### 

#### 

##### Participants.

Eighteen participants with SZ (2 female) and 14 healthy control participants (6 female) gave informed written consent to take part in this study. Participants with SZ were recruited from outpatients at the Institute of Psychiatry, Kings College London, and the Clozapine Clinic, Cambridgeshire and Peterborough NHS Foundation Trust. All were diagnosed with SZ according to DSM-IV-R criteria by an experienced psychiatrist. Of the 18 patients tested, 12 were diagnosed with paranoid SZ and the remaining six did not fall into any specific subcategory. Participants symptom severity was assessed using the Positive and Negative Symptoms Scale (PANSS) (Kay et al., 1987) within 1 week of testing. The National Adult Reading Test (NART) was used to estimate IQ in all participants (Nelson and Willison, 1991).

Three participants with SZ were unable to maintain adequate head and eye stability during the MRI scans and two were unable to perform the central fixation task, so their data have not been included. For the remaining 13 patients and 14 controls, there was no significant difference in age (patients 40 years ± 9.2, controls 33.9 years ±7.5: *t*_(25)_ = 1.881, *p* = 0.072); however, there was a significant difference in IQ (patients 98 ± 9.7, controls 111.7 ±8.9: *t*_(25)_=-3.825, *p* = 0.001; see Table 1 for patient demographics). The main results presented in Figures 3 and 4 are for this larger, unmatched group; however, given the link between IQ and strength of perceptual suppression (Melnick et al., 2013), we also provide statistical results for a smaller IQ-/age-matched group (*n* = 10; age *t*_(18)_ = 2.021, *p* = 0.058, IQ *t*_(18)_ = 1.973, *p* = 0.064). The results for the latter group strengthen the effects seen in the larger unmatched group. All procedures were approved by the University College London Research Ethics Committee. All participants had normal or corrected-to normal visual acuity.

**Table 1.** Clinical data for the full group of 13 people with schizophrenia

Diagnosis	Sex	Age	Med	Type	Dose	IQ	tPANSS	tPSS	tNSS	tGSS	tDIS	DIS
SZ	M	39	Aripiprazole	2nd	133	95	44	9	12	23	9	1
SZ	F	38	Clozapine	2nd	800	100	100	20	28	52	15	4
PS	M	42	Clozapine	2nd	750	101	59	13	14	32	9	1
PS	M	53	Clozapine	2nd	1000	89	40	12	9	19	8	2
SZ	M	36	Olanzapine	2nd	200	111	42	7	14	21	8	1
PS	M	28	Pipotiazine	1st	200	101	64	11	23	30	14	3
SZ	M	53	—	—	150	95	73	16	25	32	11	1
SZ	M	31	Clozapine	2nd	800	100	63	13	18	32	10	1
PS	F	43	Quetiapine	2nd	1400	117	55	12	17	26	11	2
PS	M	30	Clozapine	2nd	1000	106	58	12	20	26	9	1
PS	M	28	Clozapine	2nd	500	84	53	9	20	24	14	3
PS	M	49	Clozapine	2nd	1200	86	63	15	16	32	9	3
PS	M	50	Olanzapine	2nd	200	89	47	7	17	23	10	1
Mean	—	40	—	—	641	98	58.5	12	17.9	28.6	10.5	1.8
SD	—	9.2	—	—	438.9	9.7	15.8	3.7	5.3	8.4	2.4	1.1

The following information is provided: diagnosis (SZ = schizophrenia; PS = paranoid schizophrenia), medication (Med), medication type (1st = first-generation antipsychotic; 2nd = second-generation antispsychotic), medication dose (chlorpromazine equivalent in mg/d), intelligence quotient (IQ/NART score), total scores for the entire PANSS test (tPANSS), total scores for the positive symptoms of the PANSS test (tPSS), total scores for the negative symptoms of the PANSS test (tNSS), total scores for the general symptoms of the PANSS test (tGSS), scores on a cognitive factor, which overlaps heavily with the concept of disorganization syndrome (tDIS), and scores for item P2 on the PANSS test, “conceptual disorganization” (DIS).

##### fMRI stimuli and pRF mapping.

**Figure 1.**
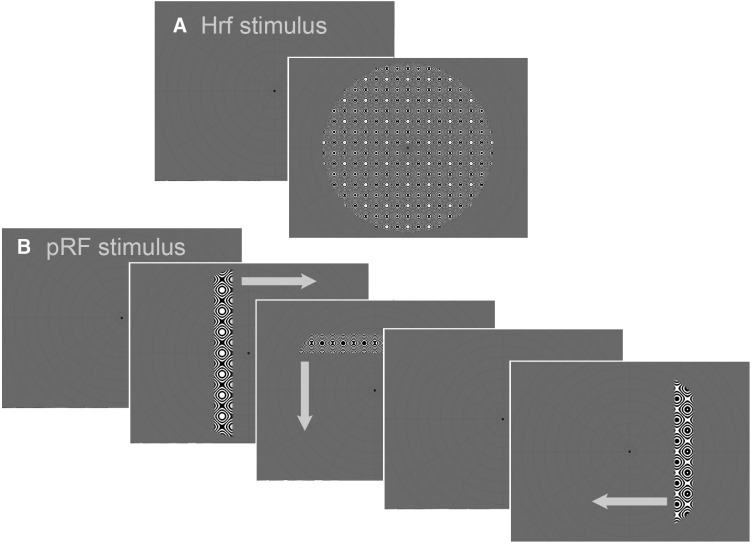
Illustration of the stimuli used for modeling the HRF (***A***) and pRF mapping (***B***). In ***A***, the full field rippling stimulus was presented briefly for 2.55 s (1 volume) and the background fixation screen then reappeared for 28.05 s (11 volumes) before another full-field stimulus was presented again. This sequence was repeated 10 times per HRF scan run. In ***B***, each image represents one volume of each block type. Only four (out of six) blocks are represented here. For each orientation/direction, the bar aperture traversed the entire field in 24 discrete steps. The third and sixth block always contained a blank period. The order of sweep directions varied between scan runs.

Each participant underwent five functional scan runs: four runs for pRF mapping and one run to estimate the hemodynamic response function (HRF). For all of these, a high-contrast dynamic “ripple” stimulus (Fig. 1*A*) was used to maximize the visual response (for further details of the stimulus, see Schwarzkopf et al. (2014)). Images were projected onto a rear-mounted screen, which was viewed via a mirror system mounted on the head coil. In this position, the full-aperture stimulus covered a circular region that subtended 9° of visual angle around fixation. All stimuli were generated in MATLAB R2012a (The MathWorks) and displayed using Psychtoolbox-3 (Brainard, 1997).

For the mapping runs, participants fixated centrally while passively viewing a moving bar aperture that exposed the dynamic rippling stimulus (Fig. 1*B*). The bar aperture subtended 1.5° and traversed the visual field in 24 discrete steps of 0.75° (1 step per fMRI image acquired). The bar was oriented horizontally or vertically and could move in either direction across the screen, making four possible sweep orientations/directions in total. Each scan run contained four sweeps of the bar (one for each orientation/direction) and two blank periods. The order of the bar orientation/direction varied across scan sessions, but the blank periods always came after the second and fourth sweep. The bar aperture was bound by the outer edge of the circular ripple pattern, where the contrast of the ripple was linearly ramped down to zero over 0.28°. For the HRF measurement, the full-aperture ripple image was briefly presented for 1 volume (2.55 s), followed by a long blank period of 11 volumes (28.05 s) (Fig. 1*A*). This sequence was repeated 10 times to complete the HRF scan run.

Participants were required to maintain central fixation throughout the pRF mapping and HRF scan runs and simultaneously perform a central fixation task: a central blue circle (diameter 0.23°) intermittently changed color to magenta for 200 ms. Participants indicated by key press every time they detected a color change. In addition, eye movements were monitored throughout the scan run using an Eyelink 1000 MRI-compatible eye tracker (http://www.sr-research.com). To further facilitate central fixation, a low-contrast radial pattern was superimposed over the entire stimulus area. This comprised 12 radial lines extending from just outside the fixation dot and 11 concentric rings centered on fixation increasing in radius with eccentricity.

##### Data acquisition and scan timing.

MRI data were acquired using a Siemens 3T TIM-Trio scanner using a 32-channel head coil. For the functional scan runs, a high-resolution EPI sequence (2.3 mm isotropic, interleaved slice order, 96 × 96 matrix, slice acquisition time 85 ms, TE 37 ms) was used to acquire 30 near axial slices positioned to optimize coverage of the occipital lobe. The front of the head coil was removed for these scans to allow participants an unrestricted view of the screen, leaving 20 receiving channels. To assess the homogeneity of the magnetic field with the front of the head coil removed, we acquired *B*_0_ field maps after the functional scan runs (double-echo FLASH sequence, short TE 10 ms, long TE 12.46 ms, 3 × 3 × 2 mm resolution, 1 mm gap). We also acquired two T1-weighted structural images, one with the front of the coil removed (MPRAGE, 1 mm isotropic voxels, 176 sagittal slices, 256 × 215 matrix, TE 2.97 ms, TR 1900 ms), which was used as an intermediate step in coregistering functional data to a second, high-resolution anatomical image acquired with the full head coil (3D MDEFT, 1 mm isotropic voxels, 176 sagittal slices, 256 × 240 matrix, TE 2.48 ms, TR 7.92 ms, TI 910 ms). The latter was used for segmentation and cortical reconstruction.

For each pRF mapping scan run, we acquired 148 volumes using a TR of 2.55 s (total duration 6 min 17 s). This included four “dummy” scans acquired at the beginning of each run to allow the brain to reach steady-state magnetization. The central blue dot was presented during the dummy scans and central fixation maintained before the first mapping stimulus appeared. Each sweep of the traversing bar stimulus or blank period lasted 61.2 s, during which time we acquired 24 volume images. For the HRF scan run, we acquired 124 volumes (including 4 dummy scans) using a TR of 2.55 s (total duration 5 min 16 s).

##### MRI data analysis.

All functional data were preprocessed using SPM8 (http://www.fil.ion.ucl.ac.uk). All functional images were intensity bias corrected using in-house software to aid automated preprocessing of the images. The dummy volumes were then discarded and the remaining images from the mapping and HRF scans were realigned and unwarped (using the *B*_0_ field maps to correct any image distortion) and coregistered to the individual's high-resolution T1 structural image acquired with the coil on, using the additional MPRAGE structural image acquired with the front of the head coil off as an intermediate step.

Freesurfer software (http://surfer.nmr.mgh.harvard.edu, version 5.0.0) was used to create 3D surface meshes of both cortical hemisphere for each individual, one for the boundary between gray and white matter and one for the outer pial boundary of the white matter. The cortical surfaces were then inflated.

All further analyses were performed using a custom MATLAB toolbox developed in-house (http://dx.doi.org/10.6084/m9.figshare.1344765) for pRF analysis and for projecting data onto the cortical surface. Data analysis was restricted to a region including the occipital, posterior temporal, and posterior parietal areas defined manually for each individual. To project functional data onto the smoothed gray/white matter surface, we determined the point midway between the gray/white and pial surfaces for each vertex on the gray/white matter boundary and used this gray matter voxel to create a functional time series for each vertex for all mapping and HRF scan runs. Linear detrending and *z*-score normalization were applied to these time series.

To estimate each individual's HRF, we averaged the signal evoked by the 10 photic bursts of the HRF scan. Outliers greater than ±1.5 SDs from the mean were excluded from the time series of each vertex. Analysis was restricted to only visually active vertices, defined by a response >1 SEM averaged over the first 5 scans after each burst. A double-gamma function was then fitted to the averaged stimulus evoked response to estimate the HRF for each hemisphere independently. There were four free parameters: the latency of the peak response and the undershoot, the peak amplitude, and the ratio of the peak and undershoot amplitudes.

For the pRF analysis, we used a forward modeling approach similar to that described by Dumoulin and Wandell (2008) to estimate the pRF parameters for each vertex independently. The pRF was initially modeled as a 2D Gaussian in visual space with four free parameters: *x* and *y* describe the pRF center position relative to the fixation point; σ (σ_1_) denotes the SD of the Gaussian, reflecting the spatial spread of the pRF (i.e., pRF size); and β (β_1_) is the response amplitude at *x*, *y*. In a subsequent analysis, we used a DoG model (based on Zuiderbaan et al., 2012) that incorporated an inhibitory surround in addition to the excitatory center. Because the DoG model is described by a combination of two Gaussians (a central positive isotropic Gaussian and a second larger negative isotropic Gaussian), there are two additional parameters in the model fit: the SD of the larger negative surround (σ_2_) (i.e., pRF surround size) and the amplitude ratio of the two Gaussians (β_2_/β_1_).

A linear overlap between the pRF model and a binary mask of the stimulus across time was used to predict the response of the neuronal population at each vertex. This predicted neuronal response was then convolved with each individual's specific HRF before optimization of the fit between this predicted neuronal response and the measured BOLD responses.

We ran a first pass coarse fit on heavily smoothed functional data [Gaussian kernel with full-width at half-maximum (FWHM) = 8.3 mm]. Using a 3D search space comprising 15 × 15 × 34 combinations of location (*x*, *y*) and pRF size (σ), we calculated the Pearson correlation between the time series at each vertex and the search grid to find the parameters with the highest correlation between observed and predicted time series (because it is based on correlation the coarse fit did not include the β parameter). All vertices in the defined occipital area were included in this initial model fit. However, vertices for which the goodness-of-fit (*R*^2^) failed to reach 0.05 in the initial coarse fit were not analyzed further. The coarse-fit parameters were then used to seed a subsequent optimization process to fit the pRF parameters to unsmoothed data at each vertex by minimizing the residual sum of squares between the predicted and observed time series. This model-fitting stage included the β parameter. We also used the coarse-fitting parameters from the standard Gaussian model to seed the optimization procedure for the DoG model. Finally, we applied a surface based smoothing kernel of 5 mm FWHM to deal with any gaps in the maps arising at vertices with poor model fits. This is particularly important for the calculation of cortical surface area and the area subtended by each face in the surface mesh in visual space. See Schwarzkopf et al. (2014) for additional details of the model-fitting procedure.

**Figure 2.**
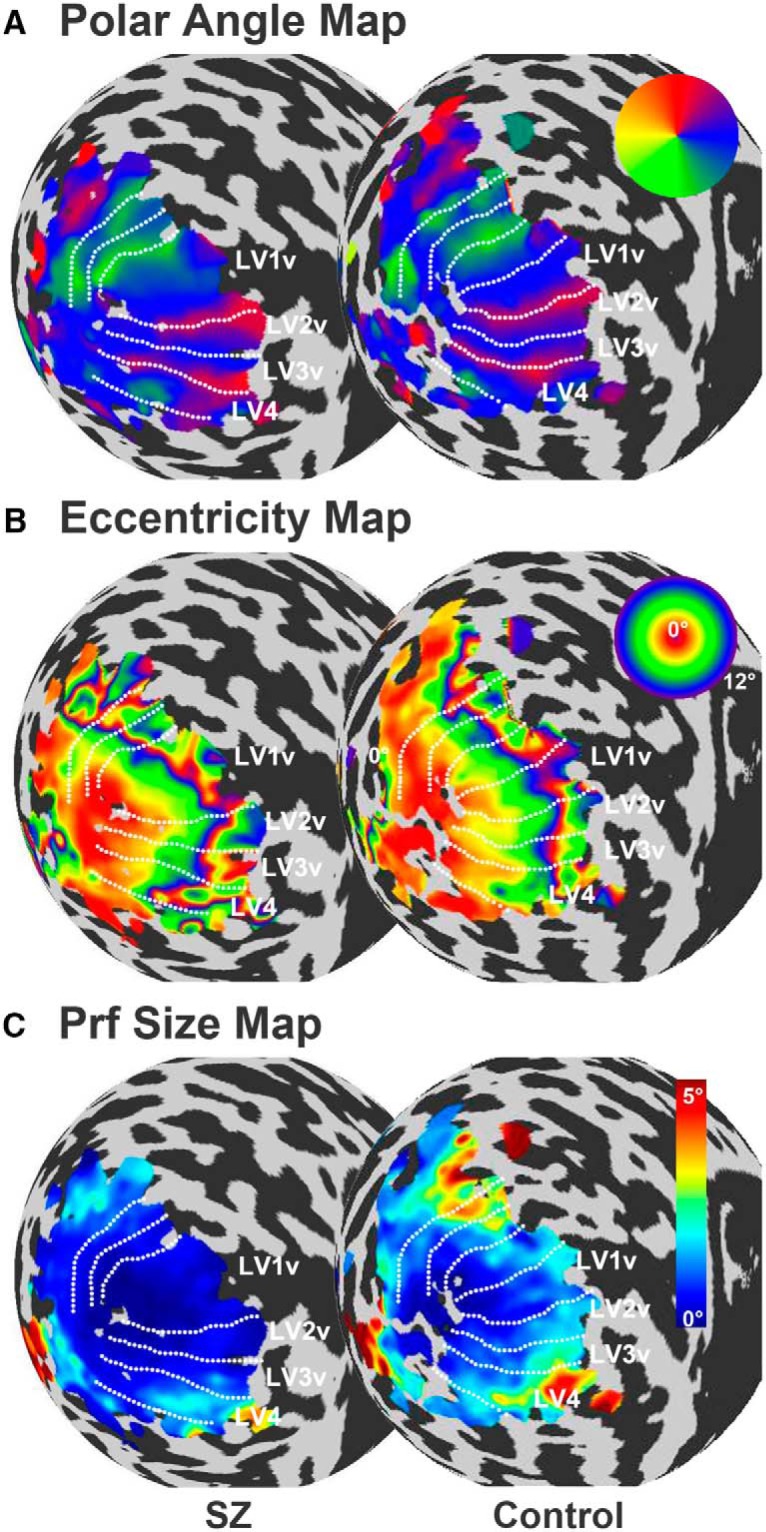
Maps for polar angle (***A***), eccentricity (***B***), and pRF size (σ) (***C***), superimposed onto an inflated spherical surface of the left hemisphere for representative individuals from each group. The borders between visual areas are defined in native space using the polar angle and eccentricity map for guidance, indicated by a dotted white contour. These boundaries have been replicated for the eccentricity and pRF size maps. Note that the eccentricity map is cyclical and wraps around at 12° eccentricity.

Visual regions were delineated manually in Freesurfer by displaying pseudocolor-coded maps of polar angle and eccentricity calculated from the pRF analysis (Fig. 2). Visual areas V1–V3 were defined using standard criteria (Sereno et al., 1995; DeYoe et al., 1996; Engel et al., 1997) and V4 was defined as a full hemifield representation adjacent to the ventral portion of V3 (Wandell et al., 2007).

To calculate the cortical magnification factor (CMF) (Harvey and Dumoulin, 2011), we divided the square root of visual area (as determined by the distances of each pRF to the pRF positions of its cortical neighbors) by the corresponding square root of the cortical surface area calculated in the same way. To measure the macroscopic surface area of these regions, we summed the area estimates of all vertices with pRF locations that fell between 2° and 7° to avoid edge artifacts.

**Figure 3.**
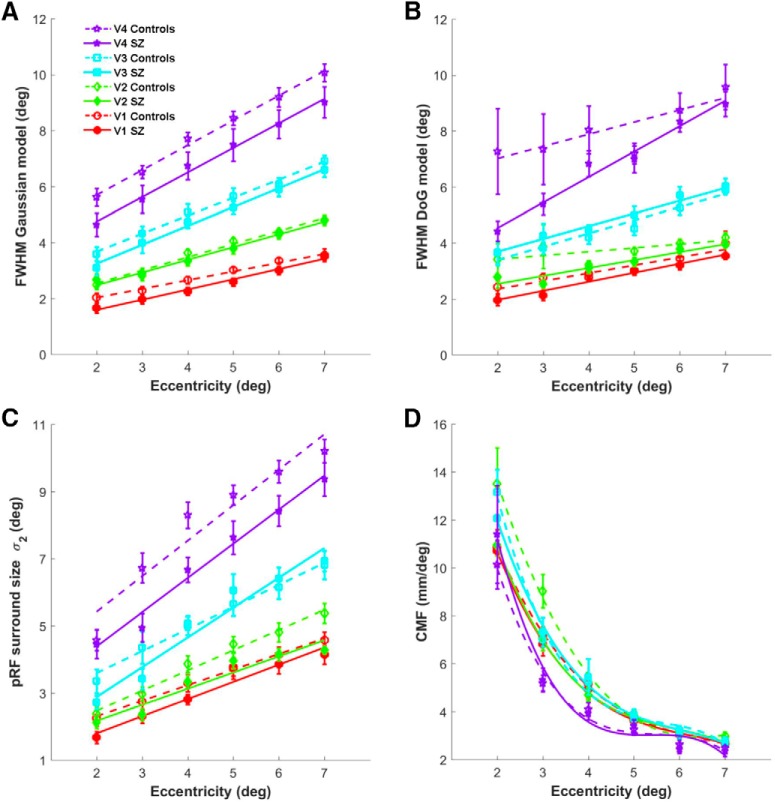
pRF size (width) and CMF averaged across participants within each group (SZ or control) and plotted against eccentricity for visual areas V1 to V4. The data presented here are for the full, unmatched, group (*n* = 13 patients, *n* = 14 controls). ***A***, FWHM reflects the width of the positive Gaussian at half the maximum response level. We used this as our measure of pRF size. The FWHM increases with eccentricity for both groups in all visual areas (as expected), but is significantly smaller in the SZ group compared with the control group in V1 and V4. For the IQ-/age-matched group, the Gaussian FWHM was significantly smaller in all visual areas (V1–V4) (data not shown in this figure). ***B***, FWHM for the central positive component of the DoG model increases with eccentricity for both groups in all visual areas, but is significantly smaller in the SZ group compared with the control group in V1 and V2. For the IQ-/age-matched group, the DoG FWHM was also significantly smaller in V1 and V2. ***C***, Sigma (σ_2_) represents pRF size (width) for the negative-surround component of the DoG model. PRF surround size also increases with eccentricity for both groups in all visual areas and is significantly smaller in the SZ group compared with the control group in V2 and V4. For the IQ-/age-matched group, the DoG surround was significantly smaller in V1, V2, and V4. ***D***, CMF decreases with eccentricity in both groups and all visual areas, but there is no significant difference between groups for any visual area. Linear regression was used to fit the pRF size by eccentricity data in ***A***–***C***. A third-degree polynomial was used to fit the CMF data. Symbols denote the group mean; solid lines represent the curve fit for the patient group; dashed lines for the control group. Error bars indicate ± 1 SEM.

For the pRF size data (σ), we subdivided the vertices into 6 1° wide eccentricity bins between 1.5° and 7.5°, thus avoiding the innermost and outermost pRFs, which were only partially mapped. For each individual, we calculated the mean pRF size (σ) for each eccentricity bin within each visual area V1–V4. We then calculated the group mean (σ) for each bin in all visual areas and fitted a linear regression to the data (Fig. 3). Any data points >2 SDs from the mean were considered outliers and were removed from the group analysis. There was no significant effect of eccentricity, visual area, pRF model, or group on the number of outliers.

For the DoG model, the Gaussian with the larger SD (the negative surround) was subtracted from the smaller Gaussian (the positive center). This results in a change to the effective positive pRF size because the width (size) of the DoG excitatory component results from a combination of the center and surround parameters and their amplitude ratio. Therefore, to allow direct comparison of the excitatory components of the two models, we followed the method of Zuiderbaan et al. (2012) and calculated the FWHM, which measures the width of the positive Gaussian at half the maximum response level. For the size of the inhibitory component of the pRF, we used the SD (σ_2_) of the negative-surround Gaussian from the DoG model.

To compare between groups, we calculated the difference in (squared) area under the curve (linear regression) fitted to the pRF size data (FWHM or σ) plotted against eccentricity (Fig. 3). To confirm that observed differences were robust, we bootstrapped the fit by resampling each group 1000 times (with replacement), refitted the curves, and recalculated the difference in squared area under the curve for each iteration. The proportion of bootstrapped differences that were opposite to the observed difference was calculated. All probability values were then corrected for multiple comparisons using the false discovery rate (FDR) with a threshold of *q* = 0.05.

## Results

For 13 patients and 14 controls, we generated polar angle and eccentricity maps to delineate the early visual areas V1–V4 (Fig. 2). Within these regions of interest, we calculated pRF size (FWHM or σ) and CMF for 6 1° wide eccentricity bins between 1.5° and 7.5°.

The standard Gaussian model fit showed that people with SZ had significantly smaller pRFs than healthy controls (Fig. 3*A*), as demonstrated by significantly less area under the curve fitted to the FWHM by eccentricity data in V1 (bootstrap test, *p* = 0.01) and V4 (*p* = 0.018), but not V2 (*p* = 0.166) or V3 (*p* = 0.062). V1 and V4 survived correction for multiple comparisons using an FDR threshold of 0.05. This result was considerably stronger for the IQ-/age-matched group, where smaller pRFs were evident in all visual areas (V1 *p* = 0.018, V2 *p* = 0.028, V3 *p* = 0.023, V4 *p* = 0.006) and all survived correction for multiple comparisons. In contrast, although there was a trend for CMF to differ between the two groups in V2 (*p* = 0.013), this did not survive correction for multiple comparisons (*p* > 0.295 in all other areas) and there were no significant differences for the IQ-/age-matched groups (*p* > 0.112 in all areas). There was also no difference between groups in the macroscopic surface area of any visual region (independent-samples *t* tests, all *p* > 0.358 for the full group, and all *p* > 0.286 for the IQ-/age-matched group). Nor were there any differences when the surface area of each region was normalized by expressing it as a percentage of overall cortical area (all *p* > 0.722 for the full group and all *p* > 0.484 for the IQ-/age-matched group). There was, however, a difference in total cortical surface area between the two groups, with the SZ group having a significant smaller overall cortical surface area compared with controls (*p* = 0.028), but this difference did not reach significance for the IQ-/age-matched group (*p* = 0.063). Previous structural imaging studies have found some support for reduced cortical surface area in SZ (Voets et al., 2008; Rimol et al., 2012).

**Figure 4.**
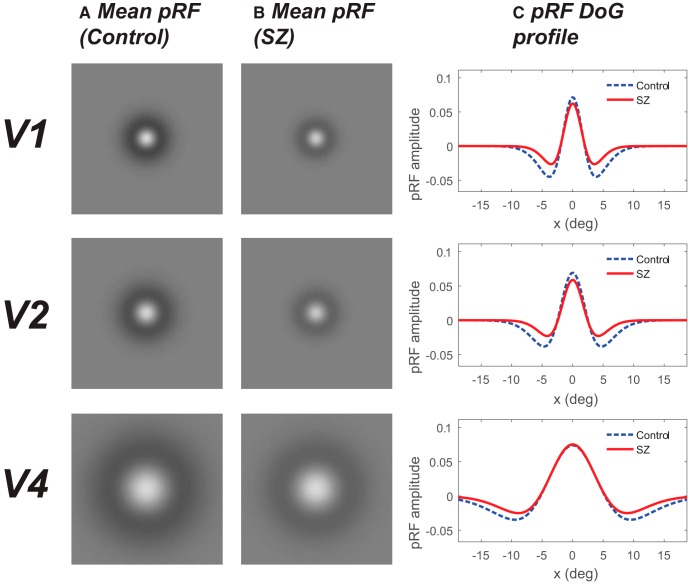
Schematic representation of the mean pRF for the DoG model in V1, V2 and V4. ***A***, ***B***, 2D plots representing the spatial extent of the mean pRF for the DoG model for controls (***A***) and patients with SZ (***B***). Bright regions signify excitation and darker regions inhibition. In all areas, patients with SZ exhibited significantly smaller inhibitory surrounds than controls. ***C***, 1D comparison of pRF profiles highlights that not only were the pRFs narrower in V1, V2, and V4, they were also shallower for people with SZ compared with controls.

We next estimated the center-surround organization of our pRFs by rerunning the pRF model using a DoG profile, which comprised an excitatory center and a larger inhibitory surround (Zuiderbaan et al., 2012). This analysis revealed that the size (σ_2_) of the negative-surround Gaussian was significantly smaller in patients with SZ in areas V1 (*p* = 0.039), V2 (*p* = 0.002), and V4 (*p* = 0.002), but not in V3 (*p* = 0.244). This difference survived correction for multiple comparisons using an FDR threshold of 0.05 in V2 and V4. For the IQ-/age-matched group, the size of the negative-surround Gaussian was significantly smaller in patients with SZ in all visual areas (V1 *p* = 0.012, V2 *p* < 0.001, V3 *p* = 0.043, V4 *p* = 0.005, and all survived correction for multiple comparisons). This effect is best seen when the pRFs are plotted in 2D (Fig. 4), making it clear that the inhibitory surround was reduced in both size and depth.

The size of the central excitatory zone, measured using the FWHM (for consistency with the standard Gaussian model), was also smaller in V1 (*p* = 0.013), V2 (*p* = 0.002), and V4 (*p* = 0.041), but not in V3 (*p* = 0.194) (Fig. 3*B*). However, only V1 and V2 survived correction for multiple comparisons. The results for the IQ-/age-matched group were similar with a significantly smaller central excitatory zone (FWHM) in V1 (*p* = 0.009), V2 (*p* < 0.001), and V4 (*p* = 0.047), but not V3 (*p* = 0.191). The results for V1 and V2 again survived correction for multiple comparisons. This apparent discrepancy with the excitatory component of the standard Gaussian model most likely arises due to a change in the central positive profile that results from subtracting the negative surround (the width of the DoG excitatory component is determined by an interaction between σ_1_ and σ_2_ and their amplitude ratio β_2_/β_1_; see Materials and Methods). Alternatively, this finding could be explained by the DoG model failing to capture center-surround pRF configurations accurately in later visual areas (V3 and beyond), an issue raised by Zuiderbaan et al. (2012). The latter might arise due to specific properties of the pRF mapping stimulus or to position scatter in later visual areas causing the center-surround configuration to be lost at the resolution of fMRI. There was certainly much greater variance in our V4 data than any other visual area (Fig. 3).

**Figure 5.**
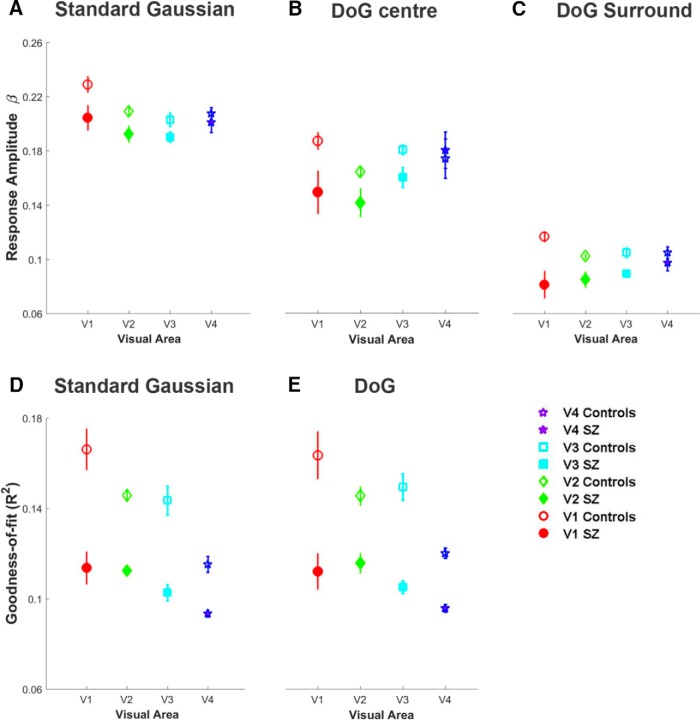
Estimates of response amplitude (β) for (***A***) the standard Gaussian model, (***B***) the central component of the DoG model, and (***C***) the surround component of the DoG model. Data have been collapsed across eccentricity with the group mean plotted for each visual area. Beta estimates were consistently lower in the SZ group for both model fits. The goodness-of-fit is plotted in (***D***), for the standard model and (***E***), for the DoG model. Model fits were consistently better for the control group, and better in V1 compared with V4 for both groups. Error bars indicate ± 1 SEM.

To determine whether our group difference in pRF size estimates were being driven by other parameters entered into the model fit, we looked for group differences in the amplitude of response to the pRF mapping stimulus (i.e., the β parameter). There was no consistent effect of eccentricity on β, so we collapsed the data across eccentricity to provide a group mean for each visual area. We observed consistently lower β values in SZ for both model fits and lower β values for the DoG model compared with the standard Gaussian model. ANOVA (with visual area (4 levels: V1 to V4) and pRF model condition (3 levels: standard Gaussian, DoG center, DoG surround) as within-subject factors) confirmed a significant main effect of group (*p* = 0.041), pRF condition (*p* < 0.001), and visual area (*p* = 0.009). For all models, the greatest difference occurred in V1, but independent *t* tests did not reveal a significant difference between groups in V1 for any model (all *p* > 0.108; Fig. 5*A–C*). The results were similar for the IQ-/age-matched group: a significant main effect of group (*p* = 0.029) and pRF condition (*p* < 0.001), but not of visual area (*p* = 0.094).

To investigate whether this group difference in β affected the quality of model fit, we then compared the goodness-of-fit (*R*^2^) of the pRF model (Fig. 5*D*,*E*) across individuals in the two groups and found that *R*^2^ was consistently higher for the healthy controls for both models. ANOVA with visual area (four levels: V1–V4) and pRF model condition (two levels: standard Gaussian and DoG) as within-subject factors confirmed a significant main effect of group (*p* < 0.001) and visual area (*p* = 0.001), but no effect of pRF model (*p* = 0.930). The results were similar for the IQ-/age-matched group: significant main effect of group (*p* < 0.001) and visual area (*p* = 0.002), but no effect of pRF model (*p* = 0.725). In a previous study (Schwarzkopf et al., 2014), we also observed better model fits (*R*^2^) with higher response amplitudes (β). However, poor model fits, which can occur due to increased noise in the data (eye movements, head movement, brain pulsatility, and optical defocus), have been shown to increase pRF size estimates for both center and surround components; therefore, it is unlikely that this is the factor driving our main effect of reduced pRF size in the patient group (Dumoulin and Wandell, 2008; Zuiderbaan et al., 2012). It should be noted, however, that *R*^2^ for both models is consistently better in V1 than V4 [paired *t*-tests comparing *R*^2^ in V1 and V4 for full group: Gaussian model (*p* = 0.001), DoG model (*p* = 0.001) and matched group: Gaussian model (*p* = 0.003), DoG model (*p* = 0.024)], which might suggest that our findings for early visual areas are more robust than those for later areas, a finding that has been noted before for the DoG model (Zuiderbaan et al., 2012).

We also investigated whether group differences in the shape and amplitude of the individually fitted HRFs could have affected our results, but we found no significant difference in the shape (area under the curve for the full group, *p* = 0.209; for the IQ-/age-matched group, *p* = 0.388) or amplitude (independent-samples *t* test for full group, *p* = 0.683; for the IQ-/age-matched group, *p* = 0.781) of the fitted HRF, consistent with a previous report that showed the hemodynamic response to be intact in medicated patients with SZ (Barch et al., 2003).

To further investigate the reliability of our data, we checked the ability of all individuals to maintain central fixation during the mapping scan runs. Eye movements were recorded throughout all scan runs and we found no significant difference in the average SD of eye position between groups for either the horizontal or vertical axes (*x*_eye_: *p* = 0.178; *y*_eye_: *p* = 0.522). We also extracted the head movement parameters calculated during the realignment stage and confirmed that there was no significant difference in the mean SD for head translation (*x*_head_: *p* = 0.228; *y*_head_: *p* = 0.088; *z*_head_: *p* = 0.098) or rotation (pitch: *p* = 0.056; roll: *p* = 0.129, yaw *p* = 0.094). There was also no significant difference in the mean SD for head translation (*x*_head_: *p* = 0.175; *y*_head_: *p* = 0.070; *z*_head_: *p* = 0.156) or rotation (pitch: *p* = 0.052; roll: *p* = 0.136, yaw *p* = 0.076) for the IQ-/age-matched group. Finally, analysis of the behavioral performance in the central fixation task also revealed no significant difference between groups (based on hit rates for the full group: *p* = 0.123 and age/IQ group *p* = 0.622).

To assess whether pRF size was associated with the severity of symptoms within the patient group, we correlated fMRI parameters with scores on the PANSS, including total PANSS score, total positive, total negative, and total general psychopathology subscale scores. We found no significant correlations between any of the PANSS scores and pRF size nor between pRF size and medication dose (the α level was set to 0.0083, reflecting Bonferroni correction for six comparisons; 6 PANSS measures assessed; Table 1). Although PANSS is considered the gold standard for measuring severity of symptoms (positive and negative), we might have found a correlation with our data if we had used one of the newer scales such as the Brief Negative Symptom Scale (BNSS; Kirkpatrick et al., 2011), which captures cognitive and negative symptoms better. We recommend using both of these scales in future studies on perception.

## Discussion

Our results indicate that the fine-grained functional architecture of early visual cortex is different in patients with SZ compared with healthy controls. A DoG model revealed that, not only was the central excitatory component of the pRF reduced in size in SZ (most reliably in V1 and V2), but the inhibitory surround was also narrower and shallower in V1, V2, and V4. Similar to a previous fMRI study (Wynn et al., 2008), we found no difference in the macroscopic organization/surface area of early visual areas between groups. Although the exact relationship between pRFs estimated using fMRI and the RFs of single neurons measured electrophysiologically remains unresolved (Logothetis et al., 2001), we do know that there is good agreement between pRF properties in humans measured using fMRI and subdural electrodes (Yoshor et al., 2007; Winawer et al., 2011) and the properties of RFs in nonhuman primates measured electrophysiologically (see Fig. 9 in Dumoulin and Wandell, 2008). Therefore, the reduction in pRF size observed here is likely to reflect a reduction in the RF size of individual neurons.

Our results are consistent with growing evidence that the fundamental pathology in SZ is a dysfunction in synaptic transmission and neuronal connectivity (Frankle et al., 2003) in association with a change in the underlying neural architecture. In support of this hypothesis, postmortem examination suggests that mean neuronal somal size is reduced in the prefrontal cortex of patients with SZ (Rajkowska et al., 1998) and this is thought to reflect a disturbance in neuronal connectivity and axonal architecture. A reduction in spine density on pyramidal cells and evidence that the cortical terminal fields are smaller than normal in the brains of patients with SZ (Lewis and Glantz, 1995) adds further weight to the hypothesis that synaptic contacts are relatively impoverished in this group (Selemon and Goldman-Rakic, 1999). Therefore, reduced pRF size in SZ may provide clues to abnormal synaptic transmission and neuronal connectivity, a feature that is difficult to assess postmortem.

It is this impaired lateral connectivity that is thought to lead to an imbalance between cortical excitation and inhibition resulting in reduced surround suppression in patients with SZ (Must et al., 2004). Surround suppression belongs to a class of neural computation known as gain control that serves to maximize the operating range of neurons, a ubiquitous feature of processing throughout the sensory cortex (Carandini and Heeger, 2011). Indeed, abnormal gain control has been proposed as the mechanism responsible for a range of visual deficits in SZ, including reduced susceptibility to contextual illusions such as the contrast–contrast phenomenon and orientation-specific surround suppression (Chubb et al., 1989; Dakin et al., 2005; Yoon et al., 2009), as well as impaired motion perception (Kim et al., 2006) and contour integration (Robol et al., 2013). Neurons in the M-pathway seem to play a central role in contrast gain control in V1. For example, M-cells in the macaque retina are prone to surround suppression (Solomon et al., 2006) and the neurons prone to the strongest surround suppression in primate visual cortex (in layers 4Cα and 4B) predominantly receive their input from M-cells (Sceniak et al., 2001). Properties of M-cells (fast response times and low spatial resolution) make them a suitable neural substrate for cortical gain control (Lennie, 1980), a process considered to reflect both intrinsic neural properties and short-range lateral interactions between neurons (Heeger, 1992). fMRI studies have also indicated a dysfunction in the magnocellular pathway in SZ (Martínez et al., 2008) and our finding of a bias toward smaller pRF size in SZ is suggestive of a loss of neurons with large receptive fields, which is consistent with a selective loss of M-cells. However, it should be noted that we did not design our stimuli to bias responses toward one cell type or other. Instead, by using a large range of SFs and relatively high contrast levels, our pRF mapping stimulus was designed to activate both magnocellular and parvocellular pathways.

NMDA receptors are also known to play a prominent role in cortical gain control within the magnocellular visual system (Kwon et al., 1992) and impaired NMDA receptor-mediated neurotransmission is thought to give rise to altered GABA receptor function, a widespread observation in SZ (Lewis, 2000). Postmortem studies show a pancortical reduction in neuronal GABA concentration in patients with SZ (Hashimoto et al., 2008), which is thought to result in abnormal gating of sensory information due to anomalous inhibitory modulation of cortical circuits (Benes and Berretta, 2001). These findings broadly support the hypothesis that reduced GABA in SZ leads to impairments in cognitive and visual tasks that involve inhibitory mechanisms as a consequence of abnormal gain control. Certainly, reduced concentration of GABA in primary visual cortex has been linked to reduced levels of gain control in SZ (Yoon et al., 2010).

Visual processing relies heavily on integration to bind together local information (e.g., brightness, color, orientation, motion) into coherent/unambiguous percepts of global structure. SZ has been linked with a deficit in such integration (Silverstein and Keane, 2011), leading to patients performing “better” than control subjects under conditions when global integration would normally interfere with responses to individual elements (Place and Gilmore, 1980; Rief, 1991; Robol et al., 2013). For example, we have shown that patient orientation discrimination thresholds for isolated Gabor targets are less elevated by the presence of disruptive clutter (“crowding”) than controls, but this effect is largely driven by patients' poor orientation discrimination of the isolated target (Robol et al., 2013). Contour integration paradigms (Field et al., 1993) have been used widely to probe the grouping deficit in SZ and indicate that patients require closer spacing of elements to detect contours (Silverstein et al., 2000; Uhlhaas et al., 2006a). These results, and related findings that patients with SZ exhibit abnormal flanker facilitation (Must et al., 2004; Kéri et al., 2005), have been taken to indicate weaker interactions between orientation detectors, most likely mediated by abnormal horizontal connectivity in V1. Certainly, broader orientation tuning has been observed in SZ (Rokem et al., 2011) and has previously been associated with reduced GABAergic inhibition (Edden et al., 2009). Reduced GABA levels have also been associated with reduced orientation-tuned surround suppression in SZ (Yoon et al., 2010). The latter could result from a reduction in either the depth of tuned suppression or an overall reduction in tuning. Therefore, it is possible that the reduction in size and depth of inhibition in the pRF surrounds that we observed in our data leads to broader orientation tuning.

In the present study, we concentrated our investigation on cortical visual areas because previous psychophysical findings have associated deficits in visual surround suppression with a predominantly cortical locus (Yoon et al., 2010; Tibber et al., 2013). However, it is possible that the change in pRF characteristics observed here are inherited from an earlier, subcortical, or even retinal origin. Certainly, changes to retinal structure and function have been documented in SZ, including loss of retinal ganglion cell axons, reduced GABA-related lateral inhibition, and dopaminergic abnormalities (for review, see Silverstein and Rosen, 2015), which may have a feedforward impact on LGN and cortical function. Imaging subcortical visual nuclei with fMRI is technically challenging (Wall et al., 2009); however, pRF mapping has provided greater precision and more detailed maps than traditional methods. Using a standard Gaussian pRF model, the spatial tuning properties of subcortical nuclei have been mapped recently in healthy individuals (DeSimone et al., 2015). The next step would be to use a DoG model to probe center-surround properties of pRFs in these areas and, if achievable, translate these methods to clinical populations such as SZ. The findings may shed light on the level at which these functional changes can be detected within the visual hierarchy. However, it should be noted that human postmortem studies have failed to find evidence for structural differences in the LGN of patients with SZ (Dorph-Petersen et al., 2009).

Among all of the basic symptoms assessed using the Bonn scale (used to identify individuals at risk of psychosis), visual distortions have the highest sensitivity for conversion to a psychotic disorder (Klosterkötter et al., 2001) and visual impairments in children are more strongly associated with later development of SZ than any other form of sensory impairment (Schubert et al., 2005). Altered neuronal density (Selemon et al., 1995), neuron number (Dorph-Petersen et al., 2007), and somal size (Rajkowska et al., 1998) all point toward some form of altered neural development. In prefrontal cortex, a reduction in mean neuronal size in the context of dramatically increased density (Rajkowska et al., 1998) suggests a subtle cellular change rather than neuronal loss, which was interpreted by the investigators as a developmental rather than a neurodegenerative change. Our findings are consistent with this hypothesis and point toward a specific change in neuronal architecture within early visual cortex that is not related to medication type or dose. Our data cannot determine at what stage in development this change occurs, but the relatively short time required to collect sufficient fMRI data to perform pRF analysis makes this a suitable technique for longitudinal tracking of changes in visual cortical architecture that could accompany the progression of SZ.

In summary, we propose that an imbalance between excitatory and inhibitory signals in SZ drives a change in the center-surround configuration of pRFs measured using fMRI. In turn, this imbalance results in broader orientation tuning and abnormal horizontal connectivity, which can ultimately explain a range of visual deficits experienced by people with SZ. Indeed, computational modeling of center-surround interactions—varying the number and strength of connections, the number of inhibitory neurons, and the time constant of GABAergic synapses—suggests that a combination of factors can result in the perceptual deficits observed (Metzner et al., 2014).
